# Fish Intake, Circulating Mercury and Mortality in Renal Transplant Recipients

**DOI:** 10.3390/nu10101419

**Published:** 2018-10-03

**Authors:** Camilo G. Sotomayor, António W. Gomes-Neto, Rijk O. B. Gans, Martin H. de Borst, Stefan P. Berger, Ramón Rodrigo, Gerjan J. Navis, Daan J. Touw, Stephan J. L. Bakker

**Affiliations:** 1 Division of Nephrology, Department of Internal Medicine, University Medical Center Groningen, University of Groningen, 9713GZ Groningen, The Netherlands; a.w.gomes.neto@umcg.nl (A.W.G.-N.); m.h.de.borst@umcg.nl (M.H.d.B.); s.p.berger@umcg.nl (S.P.B.); g.j.navis@umcg.nl (G.J.N.); s.j.l.bakker@umcg.nl (S.J.L.B.); 2 Department of Internal Medicine, University Medical Center Groningen, University of Groningen, 9713GZ Groningen, The Netherlands; r.o.b.gans@umcg.nl; 3 Molecular and Clinical Pharmacology Program, Institute of Biomedical Sciences, Faculty of Medicine, University of Chile, 8380453 Santiago, Chile; rrodrigo@med.uchile.cl; 4 Department of Pharmacy and Clinical Pharmacology, University Medical Center Groningen, University of Groningen, 9713GZ Groningen, The Netherlands; d.j.touw@umcg.nl

**Keywords:** fish intake, omega-3 polyunsaturated fatty acids, mercury, cardiovascular mortality, all-cause mortality, renal transplant recipients

## Abstract

Marine-derived omega-3 polyunsaturated fatty acids (*n*-3 PUFAs) are inversely associated with cardiovascular and all-cause mortality in renal transplant recipients (RTRs). Recommendations to increase marine-derived *n*-3 PUFAs by increasing fish intake may have a drawback in concomitant stimulation of mercury intake, which could lead to higher circulating mercury concentrations and mitigation of otherwise beneficial effects of *n*-3 PUFAs. We aimed to monitor circulating mercury concentrations, and to prospectively evaluate whether it counteracts the potential association between fish intake and cardiovascular and all-cause mortality in a cohort of RTRs (*n* = 604, 53 ± 13 years-old, 57% men) with long-term follow-up (median of 5.4 years; 121 deaths). Circulating mercury concentration (median 0.30 (IQR 0.14–0.63) µg/L) positively associated with fish intake (std. β = 0.21, *p* < 0.001). Multivariable-adjusted Cox-proportional hazards regression analyses showed that prior to, and after additional adjustment for circulating mercury concentrations, fish intake was inversely associated with both cardiovascular (HR 0.75, 95% CI 0.58–0.96; and, HR 0.75, 95% CI 0.58–0.97, respectively) and all-cause mortality (HR 0.84, 95% CI 0.72–0.97; and, HR 0.86, 95% CI 0.74–0.99, respectively). Secondary analyses accounting for marine-derived *n*-3 PUFAs intake revealed associations of similar magnitude. In conclusion, we found no evidence of a counteracting effect conferred by circulating mercury concentrations on the associations between fish and marine-derived *n*-3 PUFAs intake and the risks of cardiovascular and all-cause mortality in RTRs.

## 1. Introduction

Renal transplantation offers the highest survival benefit among existing renal replacement therapies [1]. Renal transplant recipients (RTRs), however, still carry substantially higher mortality rates compared to age-matched controls in the general population [2]. In turn, death from cardiovascular disease is the leading cause of excess premature mortality in RTRs [3].

Recent studies have provided valuable data of an inverse association between marine-derived omega-3 polyunsaturated fatty acids (*n*-3 PUFAs), and cardiovascular and all-cause mortality in the specific clinical setting of RTRs [4,5]. These findings extend to earlier studies showing that *n*-3 PUFAs derived from sea food exert beneficial effects on inflammation, fibrosis, endothelial function, lipid profile and blood pressure [6,7,8,9,10,11,12,13]. This evidence underlies why several guidelines and worldwide organizations encourage physicians to advise higher dietary fish intake in order to retrieve cardio-protective effects in diverse clinical settings [14,15,16,17,18,19], and holds the plea for further evaluation of recommendations regarding relatively higher dietary fish intake in the post-renal transplantation setting.

Controversially, however, dietary fish intake represents the major source of human exposure to organic mercury [20,21,22,23]. Methylmercury, which is the metabolized organic form of mercury that accumulates in fish tissue, is highly toxic. Although very high levels of mercury exposure are known to be acutely fatal, potential health risk conferred by chronic low-levels of mercury exposure from modest fish consumption is also of major concern [24,25,26]. Indeed, in vitro, animal-experimental and human observational studies point towards a variety of effects that together could culminate in increasing cardiovascular risk [27,28,29,30,31,32,33,34,35]. Despite being at a particularly high risk of cardiovascular disease, currently no data are available to evaluate whether mercury exposure counteracts the potential beneficial effect of a relatively higher fish intake on risk of cardiovascular mortality among RTRs.

In the current study, we sought to monitor circulating mercury by using the inductively coupled plasma mass spectrometry method in a large cohort of RTRs with long-term follow-up, and to prospectively evaluate whether it counteracts the potential inverse association of fish intake with the risks of cardiovascular and all-cause mortality. In secondary analyses, we evaluated whether circulating mercury counteracts the inverse association of marine-derived *n*-3 PUFAs intake with the risks of cardiovascular and all-cause mortality in RTRs.

## 2. Methods

### 2.1. Design and Study Population

In this prospective cohort study, all adult RTRs who survived with a functioning allograft beyond the first year after transplantation and without known or apparent systemic illnesses (i.e., malignancies, opportunistic infections), who visited the outpatient clinic of the University Medical Center Groningen (Groningen, The Netherlands) between November 2008 and March 2011, were considered eligible to participate. A total of 707 of 817 (87%) eligible RTRs signed informed consent. All patients missing dietary data or laboratory mercury measurements were excluded, resulting in 604 RTRs eligible for the analyses. The study was conducted according to the guidelines settled in the Declaration of Helsinki, and the Institutional Review Board approved the study protocol (METc 2008/186).

The primary endpoints of the current study were cardiovascular mortality, defined as death due to cerebrovascular disease, ischemic heart disease, heart failure, or sudden cardiac death according to the *International Classification of Diseases, 9th revision* (ICD-9) codes 410–447 as described previously [36,37], and all-cause mortality. Follow-up was performed for a median of 5.4 (25th–75th interquartile range (IQR) 4.9–6.0) years until September 2015. Collection of these data are ensured by the continuous surveillance system of the outpatient clinic of our university hospital. General practitioners or referring nephrologists were contacted in case the status of a patient was unknown. There was no loss due to follow-up.

All RTRs were transplanted at the University Medical Center Groningen following the establishment of standard antihypertensive and immunosuppressive therapies. Relevant characteristics including recipient age, gender, cardiovascular history, and transplant-related information were extracted from patient records. Except for discouraging excess sodium intake and encouraging weight loss in overweight individuals, no specific dietary counseling was included as a routine regimen, nor was dietary recommendation regarding fish or marine-derived *n*-3 PUFAs intake advised to the study subjects.

### 2.2. Assessment of Dietary Intake

Dietary intake was assessed with a validated semi-quantitative food frequency questionnaire (FFQ) developed and updated at the Wageningen University [38]. The FFQ examined the intake of 177 food items during the last month, taking seasonal variations into account. For each item, the frequency was documented in times per day, week, or month. The number of servings per frequency was filed in natural units (e.g., slice of bread or apple) or household measures (e.g., cup or spoon). The FFQ was self-administered and checked for completeness by a trained researcher on the day of the visit to the outpatient clinic. Inconsistent answers were verified with the patients. The results of the FFQ were converted into total energy and nutrient intake per day by using the Dutch Food Composition Table of 2006 [39].

### 2.3. Clinical Parameters

All measurements were performed during a morning visit to the outpatient clinic. Blood pressure and heart rate were determined with a semi-automatic device (Dinamap 1846, Critikon, Tampa, FL, USA), by being measured every minute for 15 min. The last three measurements were averaged, following a strict protocol as described previously [40]. Body mass index (BMI) was calculated as weight in kilograms divided by height in meters squared (kg/m^2^), and body surface area (BSA) was estimated in meters squared (m^2^) by using the universally adopted formula of DuBois and DuBois [41].

### 2.4. Laboratory Methods and Circulating Mercury Measurement

Blood was drawn after an 8–12 h fasting period, which included no medication intake. Serum high-sensitivity C-reactive protein (hs-CRP), glycated hemogloblin (HbA_1C_), triglycerides, low-density lipoprotein (LDL)-cholesterol, high-density lipoprotein (HDL)-cholesterol, and total cholesterol were measured using routine laboratory methods. Serum creatinine was determined using a modified version of the Jaffé method (MEGA AU 510, Merck Diagnostica, Darmstadt, Germany). Serum cystatin C was determined using Gentian Cystatin C Immunoassay (Gentian AS, Moss, Norway) on a Modular analyzer (Roche Diagnostics, Mannheim, Germany). According to a strict protocol, all participants were instructed to collect a 24-h urine sample the day before to their visit to the outpatient clinic. Total urinary protein concentration was determined using the Biuret reaction (MEGA AU 150, Merck Diagnostica, Darmstadt, Germany).

Mercury was measured using an in-house developed method using an inductively coupled plasma mass spectrometry method. Standard and control preparation: Mercury was measured in EDTA anticoagulated plasma. Standard plasma samples containing 0, 1, 2, 4, 6, 8, 10, 15 and 20 mcg/L mercury were made by spiking blank EDTA plasma with the same volume of a mercury standard solution (art number 1.70333.0100, Merck, Darmstadt, Germany) diluted to 1.000 mg/L with 1% nitric acid. Control samples containing 0.3, 3, 10 and 18 mcg of mercury were made by spiking blank EDTA plasma with the same volume of mercury standard solution diluted to 1.000 mg/L with 1% nitric acid. Patient sample treatment: One hundred microliters of each sample was mixed with 100 microliters of 1% nitric acid. Two hundred microliters of each sample (standard, control, patient) was mixed with 0.8 mL internal standard solution (50 mg triton X-100, 50 mg EDTA, 0.1 mg Yttrium (Merck 1.70368.0100) in 1000 mL water) and analyzed with ICP-MS on a Varian 820-MS (Varian, Palo Alto, CA, USA). With this, the method bias for 0.3, 3, 10 and 18 mcg/L was 0.1%, 1.7%, 4.7% and 6.0%. The precision was 10.4%, 7.4%, 4.5% and 6.9%. Intra-assay coefficient of variation was 9.4% and inter-assay coefficient of variation was 4.4%.

### 2.5. Calculations and Definitions

Diabetes was defined as use of antidiabetic medication, fasting plasma glucose ≥7.0 mmol/L or HbA_1C_ higher than 6.5% [42]. Renal function was assessed by the estimated glomerular filtration rate (eGFR) based on the Chronic Kidney Disease Epidemiology Collaboration Cystatin C (CKD-EPI-CysC) equation [43]. Proteinuria was defined as urinary protein excretion ≥0.5 g/24 h.

### 2.6. Statistical Analyses

Data were analyzed with SPSS version 22.0 software (SPSS Inc., Chicago, IL, USA) and R version 3.2.3 (R Foundation for Statistical Computing, Vienna, Austria). In all analyses, a two-sided *p* < 0.05 was considered significant. Data are expressed as mean ± standard deviation (SD) for normally distributed variables, and as median (IQR) for variables with a skewed distribution. Categorical data are summarized as numbers (percentage). Differences in baseline characteristics among subgroups of RTRs by categories of fish and marine-derived *n*-3 PUFAs intake were tested by ANOVA or Kruskal-Wallis test for continuous variables and by χ^2^ test for categorical variables. Univariate linear regression analyses were performed to evaluate the association of baseline characteristics with blood mercury concentrations. Residuals were checked for normality and base 2 log-transformed when appropriate. Marine-derived *n*-3 PUFAs intake was accounted as the sum of eicosapentaenoic acid (EPA, C20:5 *n*-3) and docosahexaenoic acid (DHA, C22:6 *n*-3) intake (100 mg/day) adjusted for total energy intake (kCal/day) according to the residual method [44].

To study whether fish intake was associated with the risks of cardiovascular and all-cause mortality, multivariable-adjusted Cox-proportional hazards regression analyses were performed, and Schoenfeld residuals were calculated to assess whether proportionality assumptions were satisfied. We first performed analyses in which we adjusted for general demographic characteristics (model 1; age, sex and systolic blood pressure), followed by additional adjustment for transplantation-related and post-transplantation renal function and inflammation parameters (model 2; eGFR, proteinuria status, hs-CRP, and time since transplantation), and further cumulative adjustment for glucose homeostasis (model 3; diabetes mellitus and glycemia). To avoid the inclusion of too many variables for the number of events, further models were performed with additive adjustments to model 3. We performed further adjustments for traditional cardiovascular risk factors (model 4; history of cardiovascular disease, triglycerides, and plasma LDL-cholesterol levels); and lifestyle (model 5; smoking status and alcohol use). Thereafter, we performed adjustment for circulating mercury concentrations over each one of these Cox regression models. Likewise, in secondary analyses, the aforementioned Cox-proportional hazards regression analyses with and without adjustment for circulating mercury concentrations were performed for marine-derived *n*-3 PUFAs intake instead of fish intake. We then proceeded with plotting of Kaplan-Meier curves and log-rank testing of the association of tertiles of circulating mercury concentrations with cardiovascular mortality. Finally, we performed Cox regression analyses analogues to models 1 to 5 for the association of circulating mercury concentrations with cardiovascular mortality.

## 3. Results

### 3.1. Baseline Characteristics

We included 604 RTRs. The mean age was 53 ± 13 years-old, 57% were male, and systolic blood pressure was 136 ± 17 mmHg. Patients were included at 5.7 (1.8–12.0) years after transplantation. Median fish intake was 10.7 (3.9–18.3) g/day. Marine-derived *n*-3 PUFAs intake was 103 (41–219) mg/day. Median circulating mercury concentration was 0.30 (0.14–0.63) µg/L. Baseline characteristics of the overall population and by categories of fish intake are shown in Table 1. Baseline characteristics by tertiles of marine-derived *n*-3 PUFAs intake are shown in Table S1.

Results of univariate linear regression analyses with circulating mercury concentrations as dependent variable are shown in Table 2. Circulating mercury concentrations were positively associated with fish intake (std. β = 0.12, *p* < 0.001), marine-derived *n*-3 PUFAs intake (std. β = 0.21, *p* < 0.001), alcohol consumption (std. β = 0.16, *p* < 0.001), and total cholesterol (std. β = 0.08, *p* = 0.05).

### 3.2. Prospective Analyses of Cardiovascular and All-Cause Mortality

**Figure 1:**
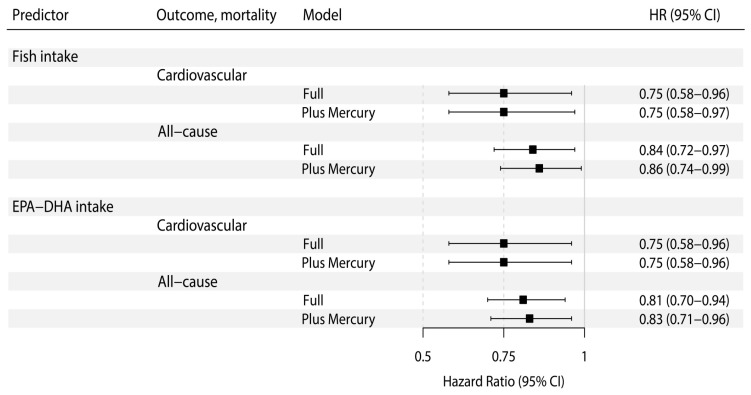
Association of fish and marine-derived omega-3 polyunsaturated fatty acids intake with cardiovascular and all-cause mortality in RTRs. Hazard ratios are calculated with adjustment for age, sex, systolic blood pressure, eGFR, proteinuria, hs-CRP, time since transplantation, plasma glucose, diabetes mellitus (Full Model), and additionally, for circulating mercury (Plus Mercury Model).

During a median follow-up of 5.4 (4.9–6.0) years, 121 (20% of the overall population) RTRs died, of which 49 (40%) deaths were due to cardiovascular causes. Prospective analyses of the association of fish intake with cardiovascular and all-cause mortality are shown in Table 3. In Cox-proportional hazards regression analyses, after adjustment for relevant covariates (i.e., age, sex, systolic blood pressure, eGFR, proteinuria, hs-CRP, time since transplantation, plasma glucose, and diabetes mellitus, according to model 3), fish intake was inversely associated with risk of cardiovascular mortality (HR 0.75; 95% CI 0.58–0.96, *p* = 0.03). This finding remained materially unaltered after further adjustment for cardiovascular- and lifestyle-related potential confounders (i.e., history of cardiovascular disease, triglycerides, and LDL-cholesterol in model 4; and, smoking status and alcohol consumption in model 5). The proportionality assumptions in the model were satisfied (Chi-squared test 0.18; *p* = 0.67). Likewise, fish intake was inversely associated with risk of all-cause mortality (HR 0.84, 95% CI 0.72–0.97, *p* = 0.02) after adjustment for relevant covariates. This finding remained materially unaltered after adjustment for cardiovascular- and lifestyle-related potential confounders. The proportionality assumptions in the model were satisfied (Chi-squared test 0.25; *p* = 0.62). In a Kaplan-Meier analysis, tertiles of circulating mercury concentrations were not associated with cardiovascular mortality (Figure S1). In addition, in Cox-regression analyses, there was no independent association of circulating mercury with cardiovascular mortality, with e.g., a HR of 1.01 (95% CI 0.48–2.14) for the third tertile of the mercury distribution compared to the first tertile after adjustment for covariates according to model 3. This finding remained materially unaltered with further adjustment for cardiovascular- and lifestyle-related potential confounders according to models 4 and 5, respectively. Further adjustment for circulating mercury over each one of the Cox regression models for the association of fish intake with cardiovascular and all-cause mortality did not materially change the associations found prior to this adjustment (see Table 3 and Figure 1).

In secondary analyses, the association of marine-derived *n*-3 PUFAs intake with cardiovascular mortality after adjustment for covariates according to model 3 was of similar magnitude (HR 0.75; 95% CI 0.58–0.96, *p* = 0.02). This finding remained materially unaltered after adjustment for cardiovascular- and lifestyle-related potential confounders according to models 4 and 5, respectively. The proportionality assumptions in the model were satisfied (Chi-squared test < 0.01; *p* = 0.99). Marine-derived *n*-3 PUFAs intake was also inversely associated with risk of all-cause mortality (HR 0.81, 95% CI 0.70–0.94, *p* = 0.01) after adjustment for covariates according to model 3. This finding remained materially unaltered after adjustment for cardiovascular- and lifestyle-related potential confounders according to models 4 and 5, respectively. The proportionality assumptions in the model were satisfied (Chi-squared test 0.11; *p* = 0.74). Additional adjustment for circulating mercury concentrations performed over each one of the Cox regression models for cardiovascular and all-cause mortality did not materially change the associations found prior to this adjustment (see Table 4 and Figure 1).

## 4. Discussion

In a large cohort of RTRs, this study shows that fish intake is positively associated with circulating mercury, and inversely associated with long-term risk of cardiovascular and all-cause mortality. We found no evidence of a counteracting effect conferred by circulating mercury on the association between fish intake and the long-term risks of cardiovascular and all-cause mortality. Likewise, we found no evidence of a counteracting effect conferred by circulating mercury on the association between marine-derived *n*-3 PUFAs intake and the long-term risks of cardiovascular and all-cause mortality in RTRs.

Recent studies provided valuable evidence of a consistent prospective independent association between marine-derived *n*-3 PUFAs and long-term survival endpoints, i.e., cardiovascular and all-cause mortality, in RTRs [4,5]. In a cohort of 1990 RTRs followed-up for a median period of 6.8 years, Eide et al. showed an inverse association between plasma biomarkers of marine-derived *n*-3 PUFAs with both risks of cardiovascular and all-cause mortality. Subsequently, in a cohort of 627 RTRs followed-up for a median period of 5.4 years, we further explored this association by providing evidence that marine-derived *n*-3 PUFAs intake is inversely associated with both risks of cardiovascular and all-cause mortality [5]. This evidence is certainly in line with, and may further support recommendations of a relatively higher dietary fish intake to retrieve cardio-protective effects in diverse clinical settings [6,7,8,9,10,11,12,13,14,15,16,17,18,19]. Remarkably, this evidence holds the plea for further studies to appropriately address concerns regarding relative harms and benefits of such dietary recommendations in the post-renal transplantation setting. On the basis that fish intake represents the major source of human exposure to organic mercury [20,21,22,23], the relevant question remains whether the benefits of fish consumption outweigh its potential harm [24,25,26,27,28,29,30,31,32,33,34,35].

Mercury is a highly reactive heavy metal with no known physiologic activity. It is methylated by organisms in marine water and concentrated through the food chain in the tissues of fish, which explains that some level of mercury is always present in marine food chain. Controversy around the balance between fish consumption benefits and mercury exposure is raised by reports stating that mercury may diminish the cardio-protective effect of fish intake [29,30]. Evidence relating mercury-induced alterations in mitochondrial Ca^2+^ homeostasis with exacerbated mercury-induced oxidative stress in kidney cells may be of particular consideration in the RTRs setting [45]. Lund et al. showed a two-fold increase in hydrogen peroxide formation in the mitochondria from kidneys of rats treated with mercuric chloride. Glutathione depletion, and inactivation of antioxidant mechanisms have also been accounted within the major mechanisms involved in mercury-induced oxidative stress in the kidney [45]. Furthermore, these mechanisms have ultimately been involved with increased arterial intima vulnerability to oxidative stress-related atherogenic processes [46,47].

Nevertheless, antioxidant dietary factors present in fish, such as selenium and vitamin E, have been proposed to subsidize the absence of a counteracting effect by mercury exposure on the benefits conferred by fish consumption [48,49,50,51]. In a nested case-control study including 33.737 men, Yoshizawa et al. showed a lack of association between total mercury exposure and the risk of coronary heart disease [52]. Ahlqwist et al. found no association between mercury exposure and the risk of myocardial infarction [53]. Hallgren et al. revealed a strong inverse association between mercury measured in erythrocytes and the risk of a first myocardial infarction [54]. Remarkably, in two U.S. cohorts, Mozaffarian et al. found no evidence of any clinically relevant adverse effect of mercury exposure on coronary heart disease, stroke, or total cardiovascular disease [50]. Our multivariable analyses that controlled for circulating mercury may, for the first time in the post-renal transplantation setting, further add to the evidence from most current epidemiologic studies that the benefits of fish consumption outweigh its potential harm conferred by concomitant mercury exposure.

The strengths of our study are as following. First, we performed complete cardiovascular and all-cause mortality endpoints evaluation despite a considerable median follow-up of 5.4 years. Second, our study comprises a large sample size of the specific clinical setting of stable outpatient RTRs. Finally, our extensively collected data allowed adjustment for several potential confounders. However, as with any observational study, unmeasured confounding may occur, despite the substantial number of potentially confounding factors we adjusted for. It should be also acknowledged that fish and marine-derived *n*-3 PUFA intake were measured using a self-reporting FFQ, which could lead to possible over- or under-reporting of dietary intake. Moreover, although circulating mercury concentration was strongly positively associated with fish intake, and it did not counteract the association of fish intake with mortality, the observed circulating mercury values were in the normal range. We cannot exclude the possibility that such counteracting effects could become perceptible over higher circulating mercury ranges. Next, in this study we did not separately account for cardiovascular complications or interventions. Developing knowledge on underlying pathways, as e.g., the ERV1/ChemR23 signaling axis, which has recently been reported to confer at least part of the protective effects of EPA supplementation on development of atherosclerotic cardiovascular disease, may provide help identifying which patients could benefit most from the protective effects of marine-derived *n*-3 PUFAs intake [55]. Finally, the population of this study consisted predominantly of Caucasian people from a single center study in The Netherlands, which calls for prudence to extrapolate our results to different populations.

## 5. Conclusions

In conclusion, in this large cohort of RTRs, fish intake positively and strongly associates with circulating mercury. Both fish and marine-derived *n*-3 PUFAs intake are inversely associated with risks of long-term cardiovascular and all-cause mortality in RTRs. The current study provides valuable data supporting that circulating mercury does not counteract these prospective associations. For the first time in the post-renal transplantation setting, our study may further add evidence to the plea that fish consumption outweighs its potential harm conferred by concomitant mercury exposure.

## Figures and Tables

**Table 1:** Baseline characteristics of the overall renal transplant recipients (RTRs) population, and by categories of fish intake.

Baseline Characteristics	Overall RTRs (*n* = 604)	Categories of Amount of Fish Intake			
		0 g/day (*n* = 118)	0–15 g/day (*n* = 241)	≥15 g/day (*n* = 245)	*p*
**Fish and marine-derived *n*-3 PUFAs intake, and circulating mercury**					
Fish intake, g/day	10.7 (3.9–18.3) ^†^	0.0 (0.0–0.0)	7.8 (4.7–10.6)	21.0 (17.0–31.9)	–
EPA-DHA intake, mg/day	103 (41–219)	20 (11–36)	70 (42–121)	240 (170–334)	<0.001
Circulating mercury concentration, µg/L	0.30 (0.14–0.63)	0.22 (0.09–0.53)	0.26 (0.13–0.62)	0.37 (0.21–0.68)	<0.001
**Demographics**					
Age, years	53 ± 13 ^‡^	51 ± 13	52 ± 13	55 ± 12	0.02
Sex (male), *n* (%)	346 (57) ^§^	70 (59)	139 (58)	137 (56)	0.82
Caucasian ethnicity, *n* (%)	602 (100)	118 (100)	239 (99)	245 (100)	0.22
**Body composition**					
Body surface area, m^2^	1.94 ± 0.22	1.96 ± 0.22	1.94 ± 0.23	1.94 ± 0.20	0.81
Body mass index, kg/m^2^	26.1 (23.2–29.3)	25.8 (23.0–29.3)	26.1 (23.14–29.4)	26.2 (23.6–29.3)	0.22
Waist circumference, cms ^a^	99 ± 14	97 ± 14	99 ± 15	99 ± 14	0.32
**Cardiovascular history**					
History of cardiovascular disease, *n* (%) ^b^	295 (49)	55 (47)	117 (49)	123 (50)	0.77
Heart rate, beats per minute ^c^	69 ± 12	68 ± 12	70 ± 12	68 ± 12	0.24
**Arterial pressure** ^d^					
Systolic blood pressure, mmHg	136 ± 17	136 ± 15	135 ± 16	137 ± 18	0.72
Mean arterial pressure, mmHg	101 ± 12	101 ± 11	100 ± 11	101 ± 13	0.60
**Antihypertensive treatment**					
Use of antihypertensives, *n* (%)	532 (88)	104 (88)	215 (89)	213 (87)	0.74
Number of antihypertensives	1.8 ± 1.1	2.0 ± 1.2	1.8 ± 1.1	1.7 ± 1.0	0.15
Use of ACE-inhibitors or ARBs, *n* (%)	198 (33)	46 (39)	73 (30)	79 (32)	0.25
Use of β-blockers, *n* (%)	383 (63)	79 (67)	153 (64)	151 (62)	0.62
Use of calcium-antagonists, *n* (%)	148 (25)	30 (25)	60 (25)	58 (24)	0.92
**Lifestyle**					
Current smoker, *n* (%) ^a^	73 (12)	15 (13)	30 (12)	28 (11)	0.92
Alcohol consumption					<0.001
None, *n* (%)	24 (4)	6 (5)	8 (3)	10 (4)	–
≤10 g/day, *n* (%)	420 (70)	91 (77)	187 (78)	142 (58)	–
>10 g/day, *n* (%)	160 (27)	21 (18)	46 (19)	93 (38)	–
Total energy intake, kCal/day	2170 ± 619	2227 ± 691	2147 ± 564	2165 ± 636	0.51
**Renal allograft function**					
Creatinine, umol/L ^e^	123 (100–158)	122 (98–166)	125 (101–157)	123 (101–156)	0.97
Cystatine-C, mg/L ^f^	1.66 (1.32–2.20)	1.70 (1.31–2.17)	1.73 (1.36–2.33)	1.60 (1.30–2.17)	0.58
eGFR, mL/min/1.73 m^2 f^	45 ± 18	46 ± 20	45 ± 18	45 ± 18	0.92
Proteinuria ≥ 0.5 g/24 h, *n* (%) ^d^	131 (22)	28 (24)	52 (22)	51 (21)	0.82
**Lipids**					
Total cholesterol, mmol/L	5.12 ± 1.11	5.12 ± 1.07	5.03 ± 1.07	5.20 ± 1.17	0.27
High-density lipoprotein-cholesterol, mmol/L ^d^	1.3 (1.1–1.6)	1.3 (1.0–1.6)	1.3 (1.0–1.6)	1.3 (1.1–1.7)	0.13
Low-density lipoprotein-cholesterol, mmol/L ^d^	2.98 ± 0.93	2.99 ± 0.86	2.94 ± 0.89	3.00 ± 1.01	0.73
Triglycerides, mmol/L	1.68 (1.24–2.30)	1.62 (1.19–2.28)	1.71 (1.27–2.37)	1.67 (1.23–2.25)	0.71
Use of statins, *n* (%)	318 (53)	59 (50)	119 (49)	140 (57)	0.19
**Diabetes and glucose homeostasis**					
Diabetes mellitus, *n* (%)	144 (24)	23 (20)	64 (27)	57 (23)	0.32
Plasma glucose, mmol/L ^e^	5.2 (4.8–6.0)	5.3 (4.8–5.9)	5.2 (4.7–6.0)	5.3 (4.9–6.1)	0.68
HbA_1C_, % ^g^	5.8 (5.5–6.2)	5.8 (5.5–6.2)	5.8 (5.4–6.3)	5.8 (5.5–6.2)	0.62
Insulin use, *n* (%)	53 (9)	6 (5)	26 (11)	21 (9)	0.20
**Inflammation and oxidative stress**					
Leukocyte count, per 10^9^/L ^d^	8.1 ± 2.6	7.8 ± 2.3	8.4 ± 2.9	7.9 ± 2.5	0.02
High-sensitivity C-reactive protein, mg/L ^h^	1.6 (0.7–4.6)	1.3 (0.5–3.6)	1.6 (0.7–4.9)	1.6 (0.8–4.7)	0.17
Malondialdehyde, µmol/L ^f^	2.62 (1.99–3.86)	2.50 (1.81–3.51)	2.44 (1.99–3.79)	2.77 (2.06–4.09)	0.10
**Renal transplantation characteristics**					
Time since transplantation, years	5.7 (1.8–12.0)	5.0 (1.7–10.6)	5.6 (2.3–11.9)	6.0 (1.4–12.2)	0.65
**Immunosuppressive therapy**					
Prednisolone dose, grams	10.0 (7.5–10.0)	10.0 (7.5–10.0)	10.0 (7.5–10.0)	10.0 (7.5–10.0)	0.62
Sirolimus or rapamune use, *n* (%)	9 (2)	3 (3)	2 (1)	4 (2)	0.42
**Type of calcineurin inhibitor**					0.78
None, *n* (%)	260 (43)	45 (38)	104 (43)	111 (45)	–
Cyclosporine, *n* (%)	242 (40)	52 (44)	95 (39)	95 (39)	–
Tacrolimus, *n* (%)	102 (17)	21 (18)	42 (17)	39 (16)	–
**Type of proliferation inhibitor**					0.13
None, *n* (%)	97 (16)	22 (19)	44 (18)	31 (13)	–
Azathioprine, *n* (%)	101 (17)	17 (14)	33 (14)	51 (21)	–
Mycophenolic acid, *n* (%)	406 (67)	79 (67)	164 (68)	163 (67)	–
Acute rejection treatment, *n* (%)	156 (26)	31 (26)	57 (24)	68 (28)	0.58

Differences in baseline characteristics among different categories of fish consumers were evaluated by using the Kruskal-Wallis test for skewed variables, ANOVA for normally distributed continuous variables and Chi-squared test for categorical data. ^†^ Median (interquartile ranges); ^‡^ mean ± standard deviation; ^§^ *n* (percentage), all such values. Data available in ^a^ 582, ^b^ 595, ^c^ 573, ^d^ 603, ^e^ 602, ^f^ 599, ^g^ 579 and ^h^ 569 patients. ACE-inhibitors, angiotensin converting enzyme inhibitors; ARBs, angiotensin II receptor blockers; DHA, docosahexaenoic acid; eGFR, estimated Glomerular Filtration Rate; EPA, eicosapentaenoic acid; HbA_1C_, glycated hemoglobin; kCal, kilocalories; *n*-3 PUFAs, omega-3 polyunsaturated fatty acids; RTRs, renal transplant recipients; Std. β, standardized beta coefficient.

**Table 2:** Results of univariate linear regression analyses with circulating mercury concentrations as dependent variable.

Baseline Characteristics	Circulating Mercury Concentration, µg/L	
	Std. β	*p*
**Fish and marine-derived *n*-3 PUFAs intake, and circulating mercury**		
Fish intake, g/day	0.21	<0.001
EPA-DHA intake, mg/day	0.21	<0.001
Circulating mercury concentration, µg/L	–	–
**Demographics**		
Age, years	–0.04	0.31
Sex (male), *n* (%)	–0.05	0.19
Caucasian ethnicity, *n* (%)	0.03	0.42
**Body composition**		
Body surface area, m^2^	0.06	0.14
Body mass index, kg/m^2^	–0.01	0.85
Waist circumference, cms ^a^	0.01	0.82
**Cardiovascular history**		
History of cardiovascular disease, *n* (%) ^b^	–0.05	0.22
Heart rate, beats per minute ^c^	–0.03	0.51
**Arterial pressure** ^d^		
Systolic blood pressure, mmHg	–0.01	0.72
Mean arterial pressure, mmHg	0.04	0.33
**Antihypertensive treatment**		
Use of antihypertensives, *n* (%)	0.02	0.67
Number of antihypertensives	<0.001	0.99
Use of ACE-inhibitors or ARBs, *n* (%)	–0.003	0.95
Use of β-blockers, *n* (%)	0.01	0.85
Use of calcium-antagonists, *n* (%)	–0.02	0.65
**Lifestyle**		
Current smoker, *n* (%) ^a^	0.02	0.57
Alcohol consumption	0.16	<0.001
None, *n* (%)	–	–
<10 g/day, *n* (%)	–	–
>10 g/day, *n* (%)	–	–
Total energy intake, kCal/day	0.02	0.64
**Renal allograft function**		
Creatinine, umol/L ^e^	0.05	0.21
Cystatine-C, mg/L ^f^	0.02	0.59
eGFR, mL/min/1.73 m^2 f^	–0.06	0.16
Proteinuria ≥0.5 g/24 h, *n* (%) ^d^	–0.04	0.39
**Lipids**		
Total cholesterol, mmol/L	0.08	0.05
High-density lipoprotein-cholesterol, mmol/L ^d^	0.05	0.23
Low-density lipoprotein-cholesterol, mmol/L ^d^	0.04	0.33
Triglycerides, mmol/L	0.02	0.62
Use of statins, *n* (%)	–0.01	0.90
**Diabetes and glucose homeostasis**		
Diabetes mellitus, *n* (%)	–0.05	0.24
Plasma glucose, mmol/L ^e^	0.001	0.98
HbA_1C_, % ^g^	–0.05	0.21
Insulin use, *n* (%)	–0.02	0.59
**Inflammation and oxidative stress**		
Leukocyte count, per 10^9^/L ^d^	–0.01	0.81
High-sensitivity C-reactive protein, mg/L ^h^	–0.01	0.78
Malondialdehyde, µmol/L ^f^	0.004	0.93
**Renal transplantation characteristics**		
Time since transplantation, years	–0.05	0.26
**Immunosuppressive therapy**		
Prednisolone dose, grams	–0.06	0.14
Sirolimus or rapamune use, *n* (%)	–0.03	0.53
**Type of calcineurin inhibitor**	0.06	0.14
None, *n* (%)	–	–
Cyclosporine, *n* (%)	–	–
Tacrolimus, *n* (%)	–	–
**Type of proliferation inhibitor**	0.03	0.52
None, *n* (%)	–	–
Azathioprine, *n* (%)	–	–
Mycophenolic acid, *n* (%)	–	–
Acute rejection treatment, *n* (%)	0.07	0.11

Univariate linear regression analyses were performed to obtain standardized βs and *p*-values for potential associations between baseline characteristics and circulating mercury concentrations. Data available in ^a^ 582, ^b^ 595, ^c^ 573, ^d^ 603, ^e^ 602, ^f^ 599, ^g^ 579 and ^h^ 569 patients. ACE-inhibitors, angiotensin converting enzyme inhibitors; ARBs, angiotensin II receptor blockers; DHA, docosahexaenoic acid; eGFR, estimated Glomerular Filtration Rate; EPA, eicosapentaenoic acid; HbA_1C_, glycated hemoglobin; kCal, kilocalories; *n*-3 PUFAs, omega-3 polyunsaturated fatty acids; RTRs, renal transplant recipients; Std. β, standardized beta coefficient.

**Table 3:** Multivariable-adjusted associations between fish intake and cardiovascular and all-cause mortality in 604 RTRs.

	Fish Intake, 10 g per day			Fish Intake, 10 g per day *		
	HR	95% CI	*p*	HR	95% CI	*p*
**Cardiovascular mortality**						
Model 1	0.82	0.64–1.03	0.09	0.82	0.65–1.04	0.10
Model 2	0.80	0.62–1.03	0.08	0.80	0.62–1.04	0.09
Model 3	0.75	0.58–0.96	0.03	0.75	0.58–0.97	0.03
Model 4	0.76	0.60–0.98	0.04	0.77	0.59–0.99	0.04
Model 5	0.76	0.58–0.98	0.04	0.76	0.58–0.99	0.04
**All-cause mortality**						
Model 1	0.88	0.76–1.01	0.06	0.90	0.78–1.03	0.12
Model 2	0.87	0.75–1.00	0.05	0.89	0.77–1.03	0.11
Model 3	0.84	0.72–0.97	0.02	0.86	0.74–0.99	0.04
Model 4	0.84	0.73–0.98	0.02	0.87	0.75–1.00	0.05
Model 5	0.84	0.73–0.98	0.02	0.86	0.74–1.00	0.05

Model 1: Age-, sex- and systolic blood pressure-adjusted. Model 2: Model 1 + adjustment for eGFR, proteinuria status, high-sensitivity C-reactive protein, and time since transplantation. Model 3: Model 2 + adjustment for history of diabetes mellitus and plasma glucose. Model 4: Model 3 + adjustment for history of cardiovascular disease, triglycerides, and LDL-cholesterol. Model 5: Model 3 + adjustment for smoking status and alcohol use. * Additional adjustment for circulating mercury concentrations.

**Table 4:** Multivariable-adjusted associations between marine-derived *n*-3 PUFAs intake and cardiovascular and all-cause mortality in 604 RTRs.

	Marine-Derived *n*-3 PUFAs Intake, 100 mg per day			Marine-Derived *n*-3 PUFAs Intake, 100 mg per day *		
	HR	95% CI	*p*	HR	95% CI	*p*
**Cardiovascular mortality**						
Model 1	0.83	0.66–1.03	0.09	0.83	0.66–1.04	0.10
Model 2	0.80	0.63–1.02	0.07	0.80	0.62–1.02	0.08
Model 3	0.75	0.58–0.96	0.02	0.75	0.58–0.96	0.02
Model 4	0.76	0.59–0.97	0.03	0.76	0.59–0.98	0.03
Model 5	0.77	0.60–0.99	0.04	0.78	0.60–1.00	0.05
**All-cause mortality**						
Model 1	0.87	0.76–0.99	0.03	0.88	0.77–1.01	0.06
Model 2	0.84	0.73–0.97	0.02	0.86	0.74–0.99	0.04
Model 3	0.81	0.70–0.94	0.01	0.83	0.71–0.96	0.01
Model 4	0.82	0.70–0.95	0.01	0.83	0.72–0.97	0.02
Model 5	0.83	0.72–0.96	0.01	0.85	0.73–0.98	0.03

Model 1: Age-, sex- and systolic blood pressure-adjusted. Model 2: Model 1 + adjustment for eGFR, proteinuria status, high-sensitivity C-reactive protein, and time since transplantation. Model 3: Model 2 + adjustment for history of diabetes mellitus and plasma glucose. Model 4: Model 3 + adjustment for history of cardiovascular disease, triglycerides, and LDL-cholesterol. Model 5: Model 3 + adjustment for smoking status and alcohol use. *Additional adjustment for circulating mercury concentrations.

## Supplementary Materials

The following are available online at http://www.mdpi.com/2072-6643/10/10/1419/s1. Figure S1: Kaplan–Meier curves and *p* value by log-rank test for cardiovascular mortality according to tertiles of circulating mercury concentrations (tertile 1: <0.185 μg/L; tertile 2: 0.185–0.485 μg/L; tertile 3: >0.485 μg/L) among RTRs. Table S1: Baseline characteristics of the overall RTRs population and by tertiles of marine-derived *n*-3 PUFAs intake.
