# Supplementary material for: Fish Intake, Circulating Mercury and Mortality in Renal Transplant Recipients
Source: Nutrients. 2018 Oct 3;10(10):1419. doi: 10.3390/nu10101419 (PMC6212909; doi:10.3390/nu10101419)
Supplement: Supplementary file 1 [file nutrients-10-01419-s001.pdf]

Fish Intake, Circulating Mercury and Mortality in Renal Transplant Recipients

Camilo G. Sotomayor [1,*], António W. Gomes-Neto [1], Rijk O. B. Gans [2], Martin H. de Borst [1], Stefan P. Berger [1], Ramón Rodrigo [3], Gerjan J. Navis [1], Daan J. Touw [4] and Stephan J. L. Bakker [1]

[1] Division of Nephrology, Department of Internal Medicine, University Medical Center Groningen, University of Groningen, 9713GZ Groningen, The Netherlands; a.w.gomes.neto@umcg.nl (A.W.G.N.); m.h.de.borst@umcg.nl (M.H.d.B.); s.p.berger@umcg.nl (S.P.B.); g.j.navis@umcg.nl (G.J.N.); s.j.l.bakker@umcg.nl (S.J.L.B.)

[2] Department of Internal Medicine, University Medical Center Groningen, University of Groningen, 9713GZ Groningen, The Netherlands; r.o.b.gans@umcg.nl (R.O.B.)

[3] Molecular and Clinical Pharmacology Program, Institute of Biomedical Sciences, Faculty of Medicine, University of Chile, 8380453 Santiago, Chile; rrodrigo@med.uchile.cl (R.R.)

[4] Department of Pharmacy and Clinical Pharmacology, University Medical Center Groningen, University of Groningen, 9713GZ Groningen, The Netherlands; d.j.touw@umcg.nl (D.J.T.)

* Correspondence: c.g.sotomayor.campos@umcg.nl; Tel.: +31-050-361-1564

SUPPLEMENTAL MATERIAL

TABLE OF CONTENTS.

Table S1 Page 3

Figure S1 Page 5

Table S1. Baseline characteristics of the overall RTRs population and by tertiles of marine-derived *n*-3 PUFAs intake.

Baseline Characteristics	Overall RTRs (*n* = 604)	Tertiles of marine-derived *n*-3 PUFAs intake			
		<54.2 mg/d (*n* = 201)	54.2–175.5 mg/d (*n* = 202)	≥175.5 mg/d (*n* = 201)	*p*
**Fish & marine-derived *n*-3 PUFAs intake, and circulating mercury**					
Fish intake, g/d	10.7 [3.9–18.3] †	0.0 [0.0–4.7]	10.4 [7.2–16.4]	23.0 [16.7–32.3]	<0.001
EPA-DHA intake, mg/d	103 [41–219]	27 [16–41]	103 [73–140]	270 [219–375]	–
Circulating mercury concentration, μg/L	0.30 [0.14–0.63]	0.18 [0.09–0.59]	0.34 [0.15–0.65]	0.36 [0.22–0.63]	<0.001
**Demographics**					
Age, years	53 ± 13 ‡	52 ± 13	53 ± 12	54 ± 12	0.12
Sex (male), *n* (%)	346 (57) §	111 (55)	114 (56)	121 (60)	0.58
Caucasian ethnicity, *n* (%)	602 (100)	201 (100)	200 (99)	201 (100)	0.14
**Body composition**					
Body surface area, m^2	1.94 ± 0.22	1.93 ± 0.23	1.94 ± 0.22	1.96 ± 0.20	0.31
Body mass index, kg/m^2	26.1 [23.2–29.3]	26.0 [22.8–29.2]	26.0 [23.3–29.4]	26.3 [24.0–29.2]	0.16
Waist circumference, cms [a]	99 ± 14	98 ± 14	98 ± 15	100 ± 14	0.31
**Cardiovascular history**					
History of cardiovascular disease, *n* (%) [b]	295 (49)	100 (50)	96 (48)	99 (49)	0.89
Heart rate, beats per minute[c]	69 ± 12	70 ± 13	68 ± 11	69 ± 12	0.13
Arterial pressure[d]					
Systolic blood pressure, mmHg	136 ± 17	135 ± 16	137 ± 17	135 ± 17	0.29
Mean arterial pressure, mmHg	101 ± 12	100 ± 11	102 ± 12	100 ± 13	0.20
Antihypertensive treatment					
Use of antihypertensives, *n* (%)	532 (88)	180 (90)	175 (87)	177 (88)	0.66
Number of antihypertensives	1.8 ± 1.1	1.9 ± 1.1	1.8 ± 1.1	1.8 ± 1.0	0.32
Use of ACE-inhibitors or ARBs, *n* (%)	198 (33)	69 (34)	57 (28)	72 (36)	0.23
Use of β-blockers, *n* (%)	383 (63)	135 (67)	126 (62)	122 (61)	0.38
Use of calcium-antagonists, *n* (%)	148 (25)	58 (29)	42 (21)	48 (24)	0.17
**Lifestyle**					
Current smoker, *n* (%) [a]	73 (12)	28 (14)	18 (9)	27 (13)	0.23
Alcohol consumption					<0.001
None, *n* (%)	24 (4)	11 (6)	8 (4)	5 (3)	–
≤10 g/d, *n* (%)	420 (70)	156 (78)	145 (72)	119 (59)	–
>10 g/d, *n* (%)	160 (27)	34 (17)	49 (24)	77 (38)	–
Total energy intake, kCal/d	2170 ± 619	2161 ± 654	2157 ± 580	2191 ± 624	0.83

**Renal allograft function**					
Creatinine, umol/L [e]	123 [100–158]	122 [96–163]	124 [99–159]	125 [103–155]	0.43
Cystatine-C, mg/L [f]	1.66 [1.32–2.20]	1.72 [1.37–2.29]	1.64 [1.29–2.33]	1.62 [1.32–2.13]	0.15
eGFR, mL/min/1.73 m^2 [f]	45 ± 18	45 ± 18	46 ± 19	45 ± 17	0.77
Proteinuria ≥0.5 g/24 h, *n* (%) [d]	131 (22)	50 (25)	37 (18)	44 (22)	0.29
**Lipids**					
Total cholesterol, mmol/L	5.12 ± 1.11	5.07 ± 1.14	5.09 ± 1.04	5.19 ± 1.16	0.53
High-density lipoprotein-cholesterol, mmol/L [d]	1.3 [1.1–1.6]	1.3 [1.0–1.6]	1.3 [1.1–1.7]	1.3 [1.1–1.7]	0.08
Low-density lipoprotein-cholesterol, mmol/L [d]	2.98 ± 0.93	2.94 ± 0.93	2.95 ± 0.87	3.03 ± 0.99	0.57
Triglycerides, mmol/L	1.68 [1.24–2.30]	1.76 [1.27–2.42]	1.66 [1.16–2.24]	1.65 [1.22–2.20]	0.26
Use of statins, *n* (%)	318 (53)	99 (49)	109 (54)	110 (55)	0.49
**Diabetes and glucose homeostasis**					
Diabetes mellitus, *n* (%)	144 (24)	47 (23)	48 (24)	49 (24)	0.97
Glucose, mmol/L [e]	5.2 [4.8–6.0]	5.3 [4.7–5.9]	5.2 [4.8–5.8]	5.3 [4.9–6.2]	0.07
HbA_{1C}, % [g]	5.8 [5.5–6.2]	5.8 [5.4–6.3]	5.8 [5.5–6.3]	5.8 [5.5–6.2]	0.78
Insulin use, *n* (%)	53 (9)	18 (9)	16 (8)	19 (10)	0.86
**Inflammation and oxidative stress**					
Leukocyte count, per 10^9/L [d]	8.1 ± 2.6	8.3 ± 2.7	8.0 ± 2.6	8.0 ± 2.5	0.28
High-sensitivity C-reactive protein, mg/L [h]	1.6 [0.7–4.6]	1.5 [0.7–3.8]	1.6 [0.6–4.2]	1.7 [0.8–5.1]	0.26
Malondialdehyde, µmol/L [f]	2.62 [1.99–3.86]	2.41 [1.90–3.47]	2.68 [1.98–3.94]	2.75 [2.06–4.14]	0.05
**Renal transplantation characteristics**					
Time since transplantation, years	5.7 [1.8–12.0]	5.1 [2.3–12.3]	5.7 [1.8–12.0]	5.9 [1.4–11.4]	0.62
Immunosuppressive therapy					
Prednisolone dose, grams	10.0 [7.5–10.0]	10.0 [7.5–10.0]	10.0 [7.5–10.0]	10.0 [7.5–10.0]	0.99
Sirolimus or rapamune use, *n* (%)	9 (2)	3 (2)	2 (1)	4 (2)	0.70
Type of calcineurin inhibitor					0.30
None, *n* (%)	260 (43)	82 (41)	79 (39)	99 (49)	–
Cyclosporine, *n* (%)	242 (40)	84 (42)	87 (43)	71 (35)	–
Tacrolimus, *n* (%)	102 (17)	35 (17)	36 (18)	31 (15)	–
Type of proliferation inhibitor					0.04
None, *n* (%)	97 (16)	41 (20)	34 (17)	22 (11)	–
Azathioprine, *n* (%)	101 (17)	29 (14)	29 (14)	43 (21)	–
Mycophenolic acid, *n* (%)	406 (67)	131 (65)	139 (69)	136 (68)	–
Acute rejection treatment, *n* (%)	156 (26)	52 (26)	52 (26)	52 (26)	1.00

Differences in baseline characteristics among different tertiles of marine-derived *n*-3 PUFAs consumers were evaluated by using the Kruskal-Wallis test for skewed variables, ANOVA for normally distributed continuous variables and Chi-squared test for categorical data. † Median [interquartile ranges]; ‡ mean ± standard

deviation; [§] n (percentage), all such values. Data available in [a] 582, [b] 595, [c] 573, [d] 603, [e] 602, [f] 599, [g] 579 and [h] 569 patients. ACE-inhibitors, angiotensin converting enzyme inhibitors; ARBs, angiotensin II receptor blockers; DHA, docosahexaenoic acid; eGFR, estimated Glomerular Filtration Rate; EPA, eicosapentaenoic acid; HbA_{1C}, glycated hemoglobin; kCal, kilocalories; *n*-3 PUFAs, omega-3 polyunsaturated fatty acids; RTRs, renal transplant recipients; Std. β, standardized beta coefficient.

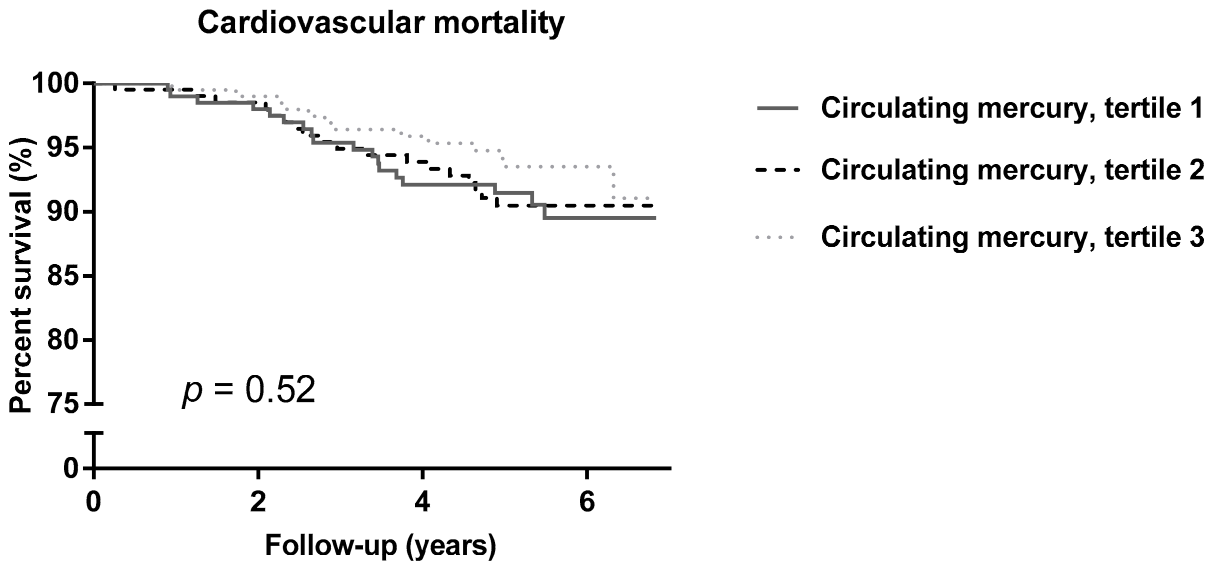

Figure S1. Kaplan–Meier curves and *p* by log-rank test for cardiovascular mortality according to tertiles of circulating mercury concentrations (tertile 1: <0.185 µg/L; tertile 2: 0.185-0.485 µg/L; tertile 3: >0.485 µg/L) among RTRs.
